# Validity of Stature-predicted Equations using Knee Height for Elderly and Mobility Impaired Persons in Koreans

**DOI:** 10.4178/epih/e2009004

**Published:** 2009-10-12

**Authors:** In Cheol Hwang, Kyoung Kon Kim, Hee Cheol Kang, Dae Ryong Kang

**Affiliations:** 1 Department of Family Medicine, Gachon University Gil Medical Center, Incheon, Korea.; 2 Department of Family Medicine, Yonsei University College of Medicine, Seoul, Korea.; 3 Graduate School of Public Health, Yonsei University, Seoul, Korea.

**Keywords:** Anthropometry, Stature estimation, Knee height, Korea

## Abstract

**OBJECTIVES:**

This study aimed to establish a stature-predicted equation using knee height, and perform a clinical validation on a Korean population.

**METHODS:**

Using nationwide data obtained from 'Size Korea 2004', a stature-predicted equation was drawn and cross validation was performed using knee height in 5,063 subjects (2,532 males, 1,785 premenopausal females, and 746 postmenopausal females) who were aged between 20 and 69 yr. The formula was then applied to an elderly group (7 males and 26 females) and a mobility-impaired group (25 males and 14 females) in a real clinical setting. A stature-predicted equation was estimated using knee height and age based on multiple linear regression analysis. Cross validation was performed using paired t-test, and validation using clinical data was performed using Wilcoxon signed rank test.

**RESULTS:**

In three groups (males, premenopausal females, and postmenopausal females), a cross validation was performed for a stature-predicted equation which was drawn using knee height and age. There were no significant differences between recorded height and estimated height in the elderly group (mean difference±interquartile range (IQR): male 0.65±4.65 cm, female -0.10±3.65 cm) and the mobility-impaired group (mean difference±IQR: male -0.23±5.45 cm, female 1.64±5.36 cm).

**CONCLUSION:**

If several limitations could be overcome, the Korean-specific equations using knee height drawn from this study could be applied to actual clinical settings with Korean elderly or mobility-impaired people.

## INTRODUCTION

Human height is generally defined as the length from the bottom of the feet to the top of the head when standing. Assessing an individual's height with accuracy plays a crucial role in determining the nutritional status of patients [1], because height is a major component of body mass index, basal metabolic rate [2], and creatinine-height index [3]. The elderly naturally show a decrease in height caused by the weakness of erector spinae muscle and reduction of water content within the intervertebral disk. In some cases vertebral compression fracture due to osteoporosis or joint contracture caused by severe osteoarthritis makes the measured height unreliable. Moreover the measuring of height itself may be impossible because of the individual's sedentary life caused by the frailty or post-stroke sequelae [4]. Therefore, it is necessary to estimate height more appropriately in theses subjects.

Estimations of height have been attempted mainly using various long bones which would not change length after discontinuation of growth. Among them, knee height and arm span were reported to be useful tools for height estimation in people whose height cannot be measured [5]. In 1985, Chumlea et al. [6] made a height estimation formula using knee height in healthy Caucasian people aged between 60 and 90 yr, and several other studies have been published on this subject since then. However, most of the estimation formulae were based on elderly people, and their fitness was assessed using cross validation [7, 8].

Therefore, we tried to make a stature-predicted equation using age and knee height in a large population of healthy Korean adults, and then confirmed the validity of this equation using cross validation. In addition to this, we attempted a clinical validation to determine whether the equation can actually be applied to elderly and mobility-impaired people.

## MATERIALS AND METHODS

### 'Size Korea 2004'

**Figure 1:**
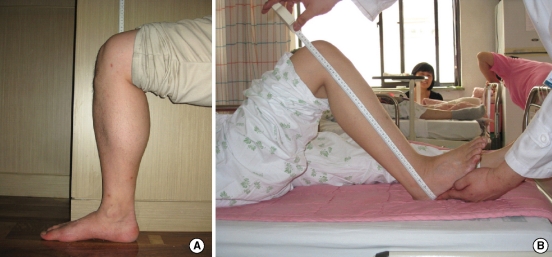
Body position for the measurement of knee height in clinical validation group (**A**, healthy elderly group; **B**, mobility-impaired group). In both groups, knee height was measured using a flexible tape while the knee and ankle joints were flexed at 90°. (**A**) It was measured in the sitting position with a tape attached to the wall, and was defined as the distance between the floor and suprapatella point. This method is identical to the one used in 'Size Korea 2004'. (**B**) In recumbent position, the measured distance is between landing point and suprapatella point from lateral side.

For this study, we used data obtained from 'Size Korea 2004' from the Korean Agency for Technology and Standards under the Ministry of Knowledge Economy. 'Size Korea 2004' is the fifth project that has measured the population of Korea, the details of which have been previously published [9]. It is one of the projects that the Korean government runs to secure high-quality data regarding the measurements of human bodies to establish a database that can be substantially used in the textile and clothing, automobile, furniture, and shoes industries. This project was conducted in every area of Korea between March 2003 and November 2004, using 15,576 Koreans, consisting of males and females who were aged between 1 and 90 years old. Within this data, this study examined 5,063 people (2,532 males and 2,531 females) aged between 20 and 69 yr. Data regarding knee height was not available for those over 70, and for those under 20, data was excluded for the reason of continual growth potential [10]. Knee height and standing height were measured using a Martin-type vertical ruler, and knee height was defined as distance from the floor to the suprapatella point in a sitting position with the knee and ankle joints flexed at 90° (Figure 1A)[11].

### Cross Validation

From the above data, age was stratified into gaps of 10 yr. In each age group, 20% of subjects were randomly selected and then served as a cross-validation group [12]. The formula was drawn from the remaining 80% of subjects. Female subjects were divided into two groups based on the cut-off value of 50 yr, which has been determined as the mean age of menopause in Korean women: premenopausal group (n=1,785) and postmenopausal (n=746) [13]. This is due to the results of epidemiological study in large groups: the decrease of height in female is accelerated around menopause [14].

### Clinical validation

For the clinical validation group, two groups were selected. The first group was comprised of 33 healthy elderly people (7 males and 26 females) aged 65 yr or older who visited the health promotion centre of Gachon University Gil Hospital in April 2008. These people had no acute illness at the time of visit that would affect measurement of height, and no past history of diseases such as vertebral compression fracture, kyphosis, scoliosis, and osteoarthritis of hip or knee. The second group was composed of 39 mobility-impaired people (25 males and 14 females) who were hospitalised in the intensive care unit or rehabilitation hospital of Yonsei University Severance hospital. These subjects had maintained a non-ambulatory life for more than one month and their height could not have been measured by usual means. Moreover, we had their past medical records including their heights prior to their non-ambulatory condition. Most of them were hospitalised due to strokes, accidents, or post-operative sequelae.

In the healthy elderly group, height was measured using an automatic digital height measuring device (InBody BSM330, Biospace, Co., Seoul, Korea). At measurement, subjects were asked to take off shoes and socks, and to touch their occipital region, back, buttock, and heel to a bar part of the device. We acquired information about height through past medical records on the mobility-impaired group. Knee height was measured using a flexible tape with sitting position in the healthy elderly group, but it was done in a recumbent position in the mobility-impaired group (Figure 1B). Knee height was defined the same way as 'Size Korea 2004' in healthy elderly group, while it was defined as the distance between landing point and suprapatella point from lateral side in the mobility-impaired group. In both groups, measurement was done with the knee and ankle joint flexed at 90°. Written consent was obtained from subjects or medical guardians before measuring the body. All the measurements were recorded to the nearest 0.1 cm, and carried out in the morning by one measurer.

### Statistical analysis

Statistical analysis was performed using SAS ver. 9.1 (SAS Inc., Cary, NC, USA). All data were expressed as mean±standard deviation except specially noted cases. In the training group, the formula estimating height based on knee height and age was drawn using multiple linear regression analysis. Cross validation was performed using paired t-test. Wilcoxon signed rank test was utilised for clinical validation, because distribution of subjects' heights and knee heights could not be considered to follow normal distribution due to the small number of subjects. A p value of less than 0.05 was considered statistically significant.

## RESULTS

From the total number of subjects, a cross-validation group was selected by an age-stratified random sampling. There were no significant differences in height and knee height as well as age between the training group and cross-validation group. For clinical validation, most of the people in the healthy elderly group were aged between 65 and 69 yr, and median age was 66.0±3.0 yr for males and 67.5±4.0 yr for females. The age distribution in the mobility-impaired group ranged from 36 to 86 yr, and median age was 59.0±21.0 yr for males and 72.5±14.0 yr for females. In all groups, male subjects had a greater measurement of height and knee height than female subjects. In female subjects, height and knee height were greater in premenopausal women than postmenopausal women (Table 1).

The formula estimating height (cm) using a multiple regression analysis in the training group was 74.63-(0.09 age)+(1.95 knee height) (R^2^=0.73, root mean square error [RMSE]=3.32) in male subjects; 66.13-(0.07 age)+(1.99 knee height) (R^2^=0.68, RMSE=2.90) in premenopausal female subjects; and 70.87-(0.14 age)+(1.96 knee height) (R^2^=0.69, RMSE=2.88) in postmenopausal female subjects. The equation using only knee height appeared to have lower R^2^ and higher RMSE compared with the one including age as a factor (not shown in table). Cross validation of drawn formula was performed with a paired t-test. Mean difference from actual height was 0.01±2.95 cm in male subjects, -0.11±2.72 cm in premenopausal female subjects, and -0.03±3.06 cm in postmenopausal female subjects. The reliability of height measurements (both estimated and actual) has been confirmed by calculating the intraclass correlation coefficient (ICC) (Table 2).

Clinical validation revealed that the mean difference from actual height was 0.65±4.65 cm in male subjects of the elderly group, -0.10±3.65 cm in female subjects of the elderly group, -0.23±5.45 cm in male subjects of the mobility-impaired group, and 1.64±5.36 cm in female subjects of the mobility-impaired group. These measurements showed no statistical significance (Table 3).

## DISCUSSION

This study is significant because it derives its stature-predicted formula using knee height and shows its clinical validity. Importantly, it was conducted using large-scale nationally representative data from the Korean population. Only a few studies have had sample sizes equivalent to the current study, such as Chumlea et al. [15] which used National Health Examination Survey data. In their study, Chumlea et al. suggested using the stature-predicted equation for mobility-impaired or handicapped people according to race and age [15]. However, there are a few differences between their study and the current one. Firstly, according to their study, the model using knee height and age in adult male subjects showed low predictability compared with the model using only knee height. In this study, however, the model using knee height and age showed a higher predictability in both male and female subjects. Because these two studies were cross-sectional designs, it is impossible to determine the exact reason. Secondly, in their study, only cross validation was performed.

Performing clinical validation is the most worthy of notice in this study. We showed that our equation is eligible in a clinical setting by the clinical validation steps. In the weight estimation area, clinical validations were already performed [16]. Weight could be measured even in patients who could not easily be measured, so long as the specific equipment was used. However, it is actually impossible to estimate height in a mobility- impaired population with any specific equipment. Moreover, according to previous studies, the use of memorised or self-reported height is also inappropriate [17]. In this study, we used data about height which was gathered by a retrospective chart analysis. Height data in medical charts were recorded when the subject was in an ambulatory status.

There are a few studies about stature estimation using knee height involving Asian populations. According to these studies, ethnicity must be considered in height estimation. In a study by Cheng et al., a stature estimated equation was assumed in Taiwanese adults aged between 25 and 85 yr [12]. They showed that ethnic differences were clarified by comparing the formulae drawn from the previous studies on different ethnicities. Myers et al. [18] noted that knee height to stature formulas were established on Japanese-Americans based on the ages between 60 to 90 yr old, and these were considered to be more specific than the Caucasian regression model developed by Chumlea and associates [6]. The only study on a Korean population is that by Han [19]. The main purpose of his study is to infer various stature prediction equations by combining knee height, total arm span, and age. Han induced various stature prediction equations in 315 persons over 60 yr old (121 males and 194 females). The more variables, the greater the prediction power. It may not be reasonable to compare this study directly to Han's because of the differences in the number of participants and of distribution. Han's conclusion that the age effect in elderly women (postmenopausal women) is greater than in elderly men is same as the result of this study (coefficient for age; male -0.15, female -0.24).

Unlike previous studies, our study drew different formulae based on whether female subjects were premenopausal or postmenopausal. In this study, a comparison of coefficient for age of each formula showed that it was 0.07 in premenopausal female subjects and 0.14 in postmenopausal female subjects. These results can be interpreted as showing that the effect of age was double in estimating height for postmenopausal women than premenopausal women, which also suggests that age-related decrease of height progresses more rapidly in postmenopausal women. Both males and females show some degree of height loss, but in the female group it accelerates at the point of menopause, usually in their 50s [14, 20]. Menopause causes many changes on body measurements. Most of the studies were based on the weight gain caused by body fat accumulation [21], but by the following it is possible to predict the change of height. First, menopause is a definite major risk factor of osteoporosis, and vertebral compression fracture caused by osteoporosis is very common in postmenopausal females [22]. Second, decrease in lean mass (or 'free fat mass') of trunk in postmenopausal female may be more menopause-related than aging effects [23]. Third, intervertebral disk space decreases prominently in the first 5-10 yr after menopause [24].

There are three limitations to our study. First, the number of subjects was not enough in the clinical validation group to apply the results to the general population. Second, it is difficult to assure reliance on the retrospective chart review, therefore a longitudinal study design is needed. Third, there were not enough efforts to minimise anthropometric measurement error. In this study, to minimise inter-observer variability and lack of dependability, the measurement was carried out by one person at the same time of day. However, it is ideally recommended in studies measuring the human body to follow a standard protocol which includes the double measurement of a sub-sample of the group under study, so that some measure of impression including % technical error measurement, R (the coefficient of reliability), and ICC can be calculated [25]. In addition, the measuring instrument or position in a clinical validation group was different from that of 'Size Korea 2004'. Flexible tape is a cheap, reliable tool that can be used for the measurement of knee height, according to Roserson's study [26], but there was no article reporting the use of a different tool in a different position as far as we know.

In conclusion, we drew a stature-predicted equation using knee height based on large-scale, nationally representative data. We showed that this equation can be applied to actual clinical settings with elderly or mobility-impaired people. This study also suggested that different equations must be applied to women according to their menopausal status. If several limitations in this study could be overcome, the equations which were drawn from the current study could be used as useful tools for the nutritional assessment of Korean subjects whose measured height is unreliable or for those whose height cannot be measured.

## Figures and Tables

**Table 1:**
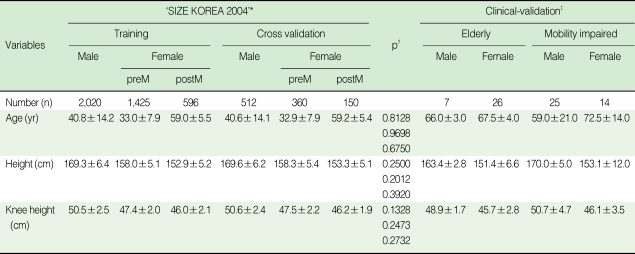
General characteristics of subjects

PreM, premenopausal; postM, postmenopausal; IQR, interquartile range. ^*^It is one of the projects that the Korean government runs to secure data about measurements of the human body. ^†^Results from two independent sample t-tests for basic characteristics of the training group and the cross validation group, in the order of male, premenopausal female, and postmenopousal female subjects. ^‡^Expressed as median±IQR by Wilcoxon signed rank test, because distribution of subjects' variables could not be considered to follow normal distribution due to the small number of subjects. Mean±standard deviation was expressed, unless noted otherwise.

**Table 2:**
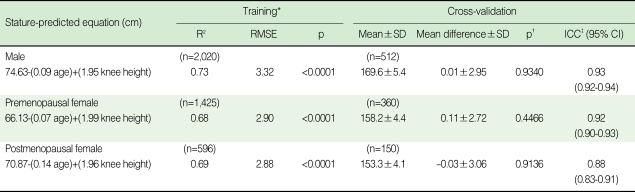
Equations for predicting stature and cross-validation

SD, standard deviation; RMSE, root mean square error; ICC, intraclass correlation coefficient. ^*^20% of subjects were selected through stratified random sampling, and multiple linear regression analysis was applied to the remaining 80% of subjects. utilising age and knee height. ^†^Paired t-test was used to compare the measured heights of the 20% of subjects selected by stratified random sampling, while the calculated heights were determined using an equation estimated from the training group. ^‡^ICC is used to calculate of the reliability of measurements between the estimated height and actual height. R^2^ means coefficient of determination on multiple regression analysis.

**Table 3:**
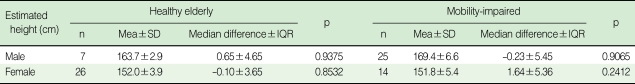
Clinical-validation in the elderly and mobility-impaired groups

SD, standard deviation; IQR, interquartile range. It was possible to confirm the difference between the heights, recorded at healthy state, with the estimated height induced from the following equation presented in Table 2. Male: 74.63-(0.09 age)+(1.95 knee height); premenopausal female: 66.13-(0.07 age)+(1.99 knee height); postmenopausal female: 70.87-(0.14 age)+(1.96 knee height). Wilcoxon signed rank test was used, as the result is not believed to demonstrate normal distribution due to a small number of study subjects.
